# Novel Respiratory Disease Diagnosis Tool: Development of an Au‐ReS_2_‐Functionalized Extended‐gate Field‐Effect Transistor‐Type Aptasensor for Simultaneous Detection of Granzyme B and Perforin

**DOI:** 10.1002/smsc.202500485

**Published:** 2026-01-14

**Authors:** Seokho Jung, Minyoung Ju, Hyunjun Park, Sunggu Kang, Jungbum Kim, Yoseph Seo, Jengmin Kang, Jong Geol Jang, Jung Hyun Choi, Dong Hyung Kim, Chulhwan Park, Min‐Ho Lee, Wonhwa Lee, Taek Lee

**Affiliations:** ^1^ Department of Chemical Engineering Kwangwoon University 20 Kwangwoon‐Ro Nowon‐Gu Seoul 01897 Republic of Korea; ^2^ Department of Chemistry Sungkyunkwan University 2066 Seobu‐Ro Jangan‐Gu Suwon 16419 Republic of Korea; ^3^ Division of Pulmonology and Allergy Department of Internal Medicine College of Medicine Yeungnam University and Regional Center for Respiratory Diseases Yeungnam University Medical Center 170 Hyeonchung‐ro Nam‐gu Daegu 42415 Republic of Korea; ^4^ Division of Biomedical Metrology Korea Research Institute of Standards and Science 267 Gajeongno Yuseong‐Gu Daejeon 34113 Republic of Korea; ^5^ Department of Biological Sciences and Bioengineering Inha University 100 Inha‐ro Michuhol‐gu Incheon 22212 Republic of Korea; ^6^ Department of Applied Measurement Science University of Science and Technology 217 Gajeong‐ro Yuseong‐gu Daejeon 34113 Republic of Korea; ^7^ School of Integrative Engineering Chung‐Ang University 84 Heukseok‐ro Dongjak‐Gu Seoul 06974 Republic of Korea

**Keywords:** dual‐biomarker detecting aptasensor, extended‐gate field‐effect transistor, granzyme B, perforin, respiratory disease diagnosis

## Abstract

Spirometry is influenced by the patient's subjective condition, which limits the reproducibility of diagnostic results despite being a key diagnostic tool for respiratory diseases. To overcome this, an extended‐gate field‐effect transistor‐type aptasensor for detecting granzyme B (GzmB) and perforin (PRF) is introduced as a proof‐of‐concept for diagnosing localized immune responses in respiratory diseases. The novel GzmB and PRF aptamers are synthesized using systematic evolution of ligands by exponential enrichment and are subsequently truncated to enhance the target‐binding affinity. Au‐ReS_2_ and the alternating current electrothermal flow technique are applied to amplify the biosensing signal and accelerate detection within 10 min, respectively. Under the 10% human serum, a linear response is observed depending on the target concentration, with the detection limits of 330 fM for GzmB and 440 fM for PRF. The targeted dual‐biomarker indicates a strong clinical correlation with bronchial conditions in chronic obstructive pulmonary disease patients. The proposed device demonstrates clear advantages in rapid, selective, and sensitive detection, suggesting its use as a preemptive diagnostic tool for respiratory diseases. This approach is expected to establish promising diagnostic strategies for early detection and therapeutic monitoring of various respiratory diseases, potentially replacing conventional spirometry.

## Graphical abstract

The simultaneous GzmB and PRF‐targeting EG‐FET‐type biosensor is developed for the diagnosis of respiratory diseases. The biosensor achieves femtomolar‐level detection of targets in human serum and reflects bronchial function according to cytotoxic immune activity in COPD patients. Therefore, the introduced system is proposed as an accurate, rapid, and sensitive diagnostic tool for respiratory diseases, capable of replacing conventional spirometry.

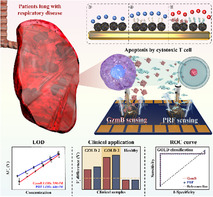

## 1 Introduction

Spirometry is commonly used as the primary diagnostic tool to assess respiratory function in patients.^[^ ^1^ ^]^ Despite its universality, this tool presents critical limitations; it is only applicable to patients who meet specific criteria and is influenced by subjective factors, such as physical and psychological conditions.^[^ ^2^ ^]^ As an alternative, noninvasive diagnostics using inflammatory factors have been proposed for the diagnosis and prognostic monitoring of respiratory diseases.^[^ ^3^ ^]^ Detection of inflammatory factors is widely applied to identify the presence of infection based on the overall level of inflammation. Such characteristics hinder the differential utilization of inflammatory markers as diagnostic targets in patients co‐infected with multiple diseases.^[^ ^4^ ^]^ In addition, their expression distribution is insignificant depending on the type of specimen, providing unclear criteria for use in practical diagnosis.^[^ ^5^ ^]^ Therefore, this diagnostic approach is unsuitable for identifying or analyzing the severity of respiratory diseases occurring at local sites.^[^ ^6^, ^7^ ^]^ Understanding the biological pathways underlying complex disease progression and developing multiplexed detection techniques are increasingly emphasized in the modern diagnostic paradigm.^[^ ^8^ ^]^ This strategy involves identifying locally expressed disease‐specific biomarkers and selecting key factors that meet the diagnostic criteria. From this perspective, identifying noninvasive biomarkers that meet these criteria and can complement existing diagnostic techniques is considered essential for the accurate diagnosis of respiratory diseases.

Cytotoxic T cells are known to play an important role in immune processes for maintaining homeostasis and resolving inflammation.^[^ ^9^ ^]^ Notably, a significant increase in cytotoxic T cell activity was observed in the small airways of patients with respiratory diseases.^[^ ^10^ ^]^ Excessive activation of cytotoxic T cells induces autoimmune diseases through apoptosis,^[^ ^11^ ^]^ which leads to tissue damage, including epithelial disruption and emphysema progression in chronic obstructive pulmonary disease (COPD), hypersensitivity pneumonitis, and idiopathic pulmonary fibrosis.^[^ ^12^, ^13^ ^]^ Granzyme B (GzmB) and perforin (PRF) have been reported to participate in sequential apoptosis pathways,^[^ ^14^ ^]^ serving as key biomarkers reflecting cytotoxic T cell‐mediated immune processes.^[^ ^10^, ^15^ ^]^ These biomolecules show different expression patterns depending on the type of respiratory disease.^[^ ^16^ ^]^ The expression of GzmB has been commonly observed in both acute and chronic respiratory diseases,^[^ ^17^, ^18^ ^]^ providing a diagnostic indicator that reflects the dual immunological responses of inflammation induction and suppression.^[^ ^19^ ^]^ PRF has been implicated in cell damage and death due to cytotoxic effects in chronic respiratory diseases.^[^ ^20^ ^]^ Interestingly, the significant upregulation of GzmB and PRF observed in the peripheral blood of patients with respiratory disease suggests that these biomarkers can indicate both immune responses to respiratory disease and nonspecific tissue damage resulting from chronic inflammation, regardless of the sampling site.^[^ ^21^, ^22^ ^]^ Therefore, monitoring GzmB and PRF may provide attractive insights into predicting the pathological status of localized respiratory diseases.

**Figure 1:**
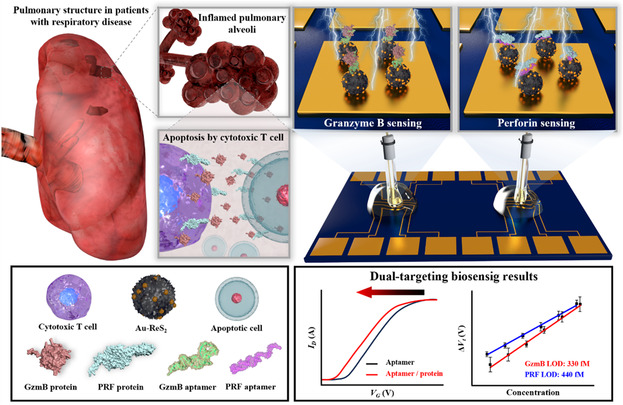
Schematic illustration of a fabricated dual‐target detectable EG‐FET‐type aptasensor for respiratory disease diagnosis.

In this study, an extended‐gate field‐effect transistor (EG‐FET) biosensor that detects GzmB and PRF is introduced as a novel diagnostic system capable of responding preemptively to respiratory diseases. The sensing membrane was configured with a micro‐sized dual electrode for simultaneous detection of biomarkers. Au‐ReS_2_ functionalization on the electrode surface was used as a strategy for sensitive detection of low‐abundance biomarkers present in clinical environments. GzmB and PRF aptamers were synthesized through the systematic evolution of ligands by exponential enrichment (SELEX) process and were truncated to enhance their binding affinity toward targets. The alternating current electrothermal flow (ACEF) technique was applied to accelerate the target detection within 10 min. The performance of the fabricated sensor was evaluated in a similar clinical environment, and its practical applicability was discussed by electrical measurements of GzmB and PRF in clinical samples from patients with COPD. A schematic illustration of the proposed respiratory disease diagnostic system was presented in **Figure** **1**.

## 2 Results and Discussion

### 2.1 Binding Characteristics of Synthesized Aptamers

**Figure 2:**
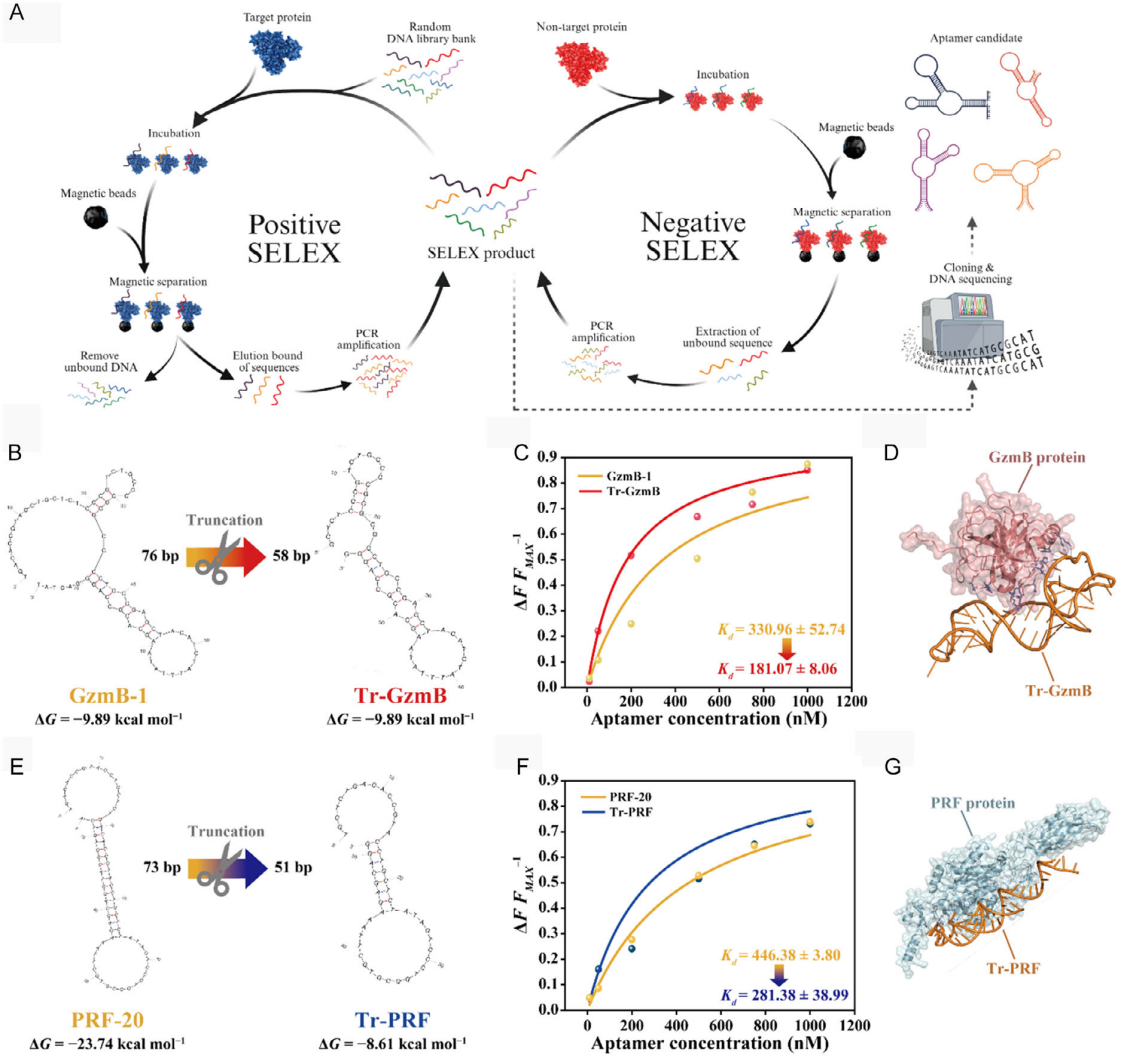
A) Schematic illustration of the SELEX process. B) 2D structure and C) binding affinity test (*n* = 3) of the GzmB aptamer with and without truncation. D) Docking simulation results between the Tr‐GzmB and GzmB. E) 2D structure and F) binding affinity test (*n* = 3) of the PRF aptamer with and without truncation. G) Docking simulation results between the Tr‐PRF and PRF. Illustrations in (A) were created with BioRender.com and used with permission.

Aptamers, composed of single‐stranded nucleic acids, serve as selective bioprobes for specific targets and offer several advantages over antibodies, including lower immunogenicity, superior stability, and cost‐effective synthesis.^[^ ^23^ ^]^ Notably, their application as promising bioprobes has been demonstrated for targeting chemicals, toxic molecules, and low‐molecular‐weight biomolecules that are challenging for antibodies.^[^ ^24^, ^25^ ^]^ The synthesis of aptamers specific to GzmB and PRF was performed by positive SELEX and negative SELEX (**Figure** **2**A). The final products after the 10th round of SELEX exhibited specific binding to the target proteins (Figure S1A,B, Supporting Information). Aptamers form divergent secondary structures depending on diluted environments such as ionic strength, salt concentration, and temperature.^[^ ^26^ ^]^ The low Gibbs free energy (Δ*G*) indicates the structural stability of an aptamer and contributes to the reproducible operation, ensuring the uniform quality.^[^ ^27^ ^]^ Based on the Δ*G* values, two aptamers were selected as candidates for each target (Table S1, Supporting Information). The dissociation constant (*K*_*d*_) of the aptamers was calculated using Equation (1).^[^ ^28^ ^]^

(1) $$\theta =\frac{\gamma -\gamma F}{\gamma R-\gamma F}$$

Here, *θ* is the proportion of aptamers bound to the target, *γ* is the equilibrium fluorescence intensity, and *γF* and *γR* are the fluorescence intensities in the absence and at a saturation concentration of the aptamer, respectively. The *K*_*d*_ values of GzmB‐1 and GzmB‐17 were 330.96 and 355.24 nM, respectively. In addition, PRF‐5 and PRF‐20 showed *K*_*d*_ values of 669.68 and 446.38 nM, respectively. A lower *K*_*d*_ value indicates the enhanced stability of the probe‐target binding complex.^[^ ^29^ ^]^ Thus, GzmB‐1 (Figure S2A, Supporting Information) and PRF‐20 (Figure S2B, Supporting Information) were finally selected owing to their superior target binding affinity.

The structural features of aptamers enable them to selectively recognize specific targets through hydrophobic interactions, electrostatic attractions, hydrogen bonding, van der Waals forces, shape complementarity, and base stacking.^[^ ^30^ ^]^ The DNA library used for the SELEX process contains predefined primer binding sites for amplification via polymerase chain reaction (PCR). These regions are unlikely to participate in target binding, and thus, truncating these sequences is used as a strategy to enhance the synthetic efficiency, cost‐effectiveness, and target binding affinity of aptamers.^[^ ^31^, ^32^ ^]^ The truncated aptamers exhibited molecular weights reflecting the shortened fragment length (Figure S3A,B, Supporting Information). Pre‐ and post‐truncated aptamers for GzmB (Figure S3C, Supporting Information) and PRF (Figure S3D, Supporting Information) exhibited highly similar spectral profiles with B‐form DNA secondary structure characteristics, indicating the structural preservation.^[^ ^33^ ^]^ The truncated (Tr)‐GzmB (Figure 2B) showed ≈43% lower *K*_*d*_ value than GzmB‐1 (Figure 2C). In the binding prediction results of Tr‐GzmB with GzmB (Figure 2D), the selected model with a confidence score of 0.9539 showed a docking score of −301.536 and ligand root mean square deviation (RMSD) of 36.02 Å, suggesting a high probability of the binding event (Table S2, Supporting Information). The Tr‐PRF based on PRF‐20 (Figure 2E) showed a decreased *K*_*d*_ value by ≈37% (Figure 2F). The proper target docking simulation model of Tr‐PRF (Figure 2G) indicated 0.9768, −336.92, and 86.67 of confidence level, docking score, and ligand RMSD, respectively (Table S3, Supporting Information). The enhanced binding performance of truncated aptamers was cross‐validated by dual‐prism solution‐immersed silicon (DP‐SIS) sensor measurements (Figure S4A, Supporting Information). Here, changes in the polarization state of reflected light are expressed as variations in the amplitude ratio (*ψ*) based on the principle of ellipsometry, which is correlated with alterations in the thickness at the sensor surface. The truncated aptamers for GzmB (Figure S4B, Supporting Information) and PRF (Figure S4C, Supporting Information) showed higher Δ*ψ* values than their corresponding full‐length counterparts (Table S4, Supporting Information), indicating enhanced binding events between the aptamers and their target proteins.^[^ ^34^ ^]^ Therefore, Tr‐GzmB and Tr‐PRF were applied to achieve sensitive target detection in the construction of subsequent biosensing platforms.

### 2.2 EG‐FET Biosensing System Construction

Transition metal dichalcogenide (TMD) is used as a biosensing signal amplification strategy due to its excellent electronic properties.^[^ ^35^, ^36^ ^]^ Among them, ReS_2_ offers abundant active sites based on its high surface area to volume ratio, strong anisotropy, excellent interlayer independence, and rapid electron transfer characteristics induced by Re–Re metallic bonding.^[^ ^37^, ^38^ ^]^ The complex formation of Au and TMD promotes electron transfer at the metal–semiconductor interface, thereby effectively improving electrical conductivity.^[^ ^39^, ^40^ ^]^ These properties suggest that Au‐ReS_2_ composites can achieve effective biosensing signal amplification via a similar synergistic mechanism. Furthermore, this strategy enables bioprobe immobilization using Au—S bonds that are free from the *N*‐hydroxysuccinimide (NHS) ester coupling method,^[^ ^41^ ^]^ which is commonly used for surface modification of TMDs. ReS_2_ and Au‐ReS_2_ showed no aggregation in the aqueous solution, and colorimetric differences were confirmed with the naked eye (Figure S5A, Supporting Information). The synthesis of Au on the ReS_2_ nanoparticles (NPs) resulted in surface plasmon resonance, which enhanced the extinction of Au‐ReS_2_ throughout the visible light spectrum (Figure S5B, Supporting Information).^[^ ^42^ ^]^ ReS_2_ exhibited an urchin‐like structure (Figure S6A, Supporting Information), and the presence of Au NPs was confirmed at Au‐ReS_2_ (Figure S6B, Supporting Information). Unique interplanar distances of Au and ReS_2_ were observed in Au‐ReS_2_ as 0.611 and 0.23 nm, respectively (Figure S6C, Supporting Information).^[^ ^43^, ^44^ ^]^ The Au‐ReS_2_ synthesis was verified by the distribution of the constituting elements (Figure S6D–F, Supporting Information). In both ReS_2_ and Au‐ReS_2_, the characteristic peaks corresponding to the intrinsic crystal planes of ReS_2_, including (100), (200), and (−122), were observed, whereas the distinctive peak of Au, including (111), (200), (220), (311), and (222), appeared exclusively in Au‐ReS_2_ (Figure S7A, Supporting Information).^[^ ^44^, ^45^ ^]^ The X‐ray photoelectron spectroscopy (XPS) analysis of Re^4+^ showed peaks at 41.8 and 44.2 eV at 4f_7/2_ and 4f_5/2_, respectively, in ReS_2_ (Figure S**7**B, Supporting Information).^[^ ^46^ ^]^ These peaks were identified at 41.9 and 44.3 eV in Au‐ReS_2_, and this positive shift in binding energy is attributed to the n‐doping effect, which shifts the Fermi level.^[^ ^47^ ^]^ The S peaks of 2p_3/2_ and 2p_1/2_ observed in ReS_2_ were 162.5 and 163.9 eV, respectively, and in Au‐ReS_2_, they were 162.3 and 163.5 eV, respectively, showing a decrease in binding energy due to Au functionalization (Figure S7C, Supporting Information).^[^ ^43^ ^]^ The Au peaks of 4f_7/2_ and 4f_5/2_ were indicated only in Au‐ReS_2_ at 83.9 and 87.6 eV, respectively (Figure S7D, Supporting Information).^[^ ^48^ ^]^

**Figure 3:**
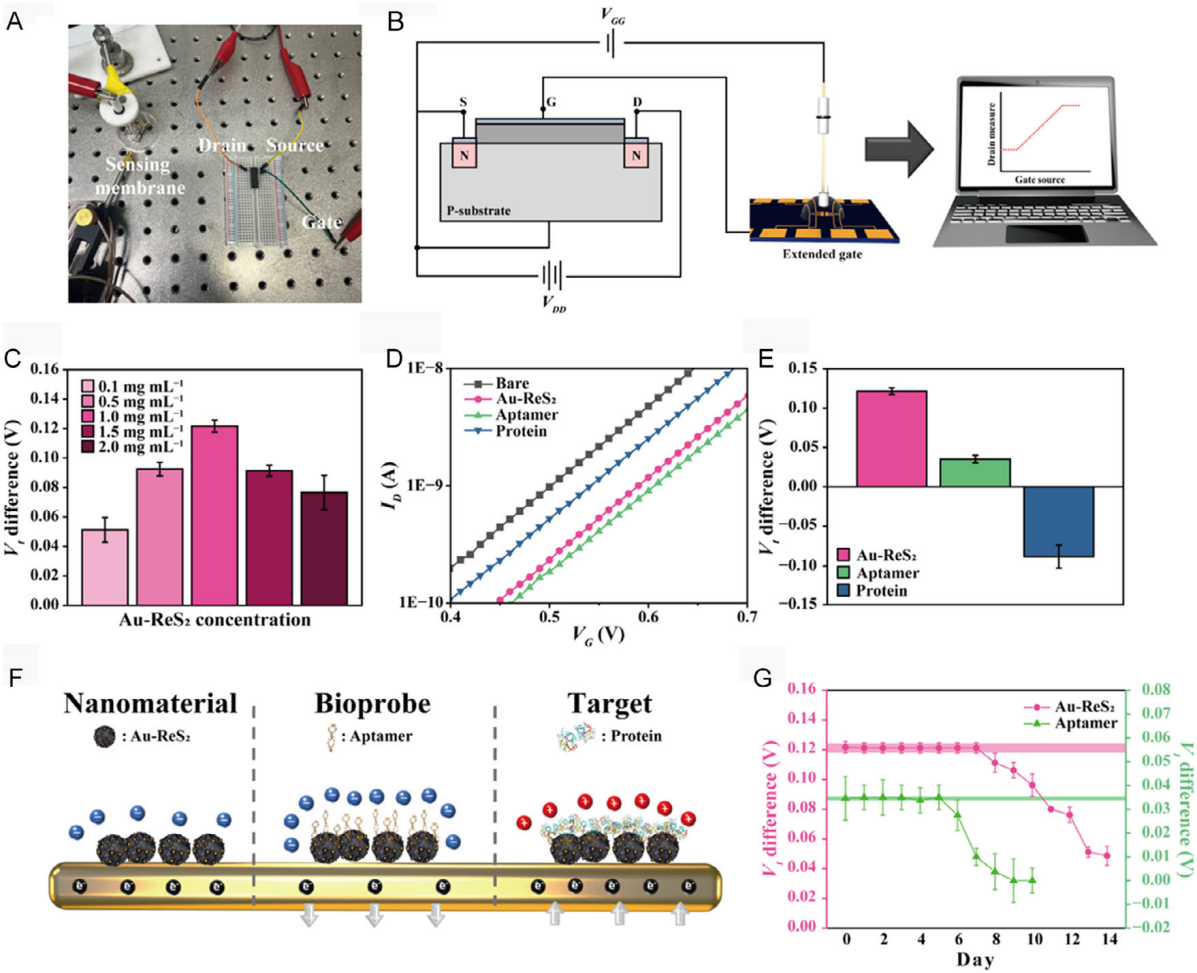
A) Real photo and B) circuit diagram of a developed EG‐FET biosensing system. C) *V*_*t*_ difference according to the Au‐ReS_2_ concentrations. D) *I*_*D*_–*V*_*G*_ curves and E) *V*_*t*_ values at each sensing membrane functionalization step. F) Schematic illustration of changes in electron density at sequential biosensing structure construction. G) Daily stability test of biosensing structure functionalized with Au‐ReS_2_ and aptamer. Electrical measurements were repeated to ensure reproducibility and reliability (*n* = 8). The pink and green boxes represent the 95% confidence intervals based on the measurement signal at 0 day, with Au‐ReS_2_ and aptamer immobilization, respectively.

In the FET‐based biosensing platform fabrication, the gate was extended by connecting to a sensing membrane (**Figure** **3**A). The measurement system was constructed to faithfully detect the electrical characteristics of a target biomarker (Figure 3B). Au‐ReS_2_ generated the largest electrical signal change at a concentration of 1 mg mL^−1^, providing optimal conductivity to the sensing membrane (Figure 3C). The decrease in *V*_*t*_ at higher concentrations than 1 mg mL^−1^ suggested that the high concentration resulted in the removal of aggregated samples during passivation.^[^ ^49^ ^]^ The 100 nM concentration of aptamer induced the most dramatic increase in signal and was selected as the optimal condition (Figure S8, Supporting Information). Electrons act as the main charge carriers in an n‐type FET. The occurrence of the n‐charge doping effect shifts the threshold voltage (*V*_*t*_) in a positive direction.^[^ ^50^, ^51^ ^]^ The Au‐ReS_2_‐functionalized sensing membrane possessed negatively charged characteristics,^[^ ^52^, ^53^ ^]^ shifting the drain current (*I*_*D*_)‐gate voltage (*V*_*G*_) curves in the positive direction (Figure 3D). Aptamers with negative charge due to their phosphate backbones showed a similar shifting trend. Proteins have intrinsic isoelectric points (pI), and their relative electrical characteristics are determined by the pH of the diluted solution.^[^ ^54^ ^]^ In electrical biosensing, this phenomenological understanding is interpreted as a key factor in the binding of proteins with amphoteric charge properties during the detection step. The pI values of GzmB and PRF are 9.56 and 7.90, respectively, which caused a positive charge doping effect under measurement conditions, shifting the *I*_*D*_–*V*_*G*_ curves negatively. The *V*_*t*_ difference showed the sequential modification of the sensing membrane (Figure 3E). The electron density in the channel was influenced according to the charge of the material introduced on the sensing surface (Figure 3F). The sensing membrane modification was cross‐validated using electrochemical measurements (Figure S9A, Supporting Information). The charge transfer resistance (*R*_*ct*_) of the electrochemical impedance spectroscopy (EIS) measurement indicates the redox reaction that occurs between the sensing membrane and the buffer.^[^ ^55^ ^]^ Functionalization with Au‐ReS_2_ and aptamer induced changes in *R*_*ct*_ by enhancing conductivity and inhibiting redox reactions, respectively (Figure S9B, Supporting Information). The electrochemical results by the positively charged target were consistent with the previously confirmed electrical signal trends, supporting the reproducibility and reliability of the biosensor fabrication. In the surface characterization of the functionalization step, Au‐ReS_2_ showed a similar morphological property to the scanning electron microscopy (SEM) analysis, and subsequent sequential depositions of biomolecules were confirmed by changes in surface properties (Figure S10A, Supporting Information). The overall increase in vertical distance (VD), root mean square (RMS), and roughness average at each step indicated the sequential construction of the biosensing structure (Figure S10B, Supporting Information). The linear lengths of GzmB and PRF aptamers were calculated to be ≈32.48 and 28.56 nm, respectively.^[^ ^56^ ^]^ The increase in VD of ≈16.35 nm by the aptamer was attributed to the structural folding. Due to post‐translational modifications that affect the biomolecular properties of proteins, the approximate prediction of the actual protein size was limited using its molecular weight.^[^ ^57^ ^]^ Therefore, protein capturing by the aptamer was confirmed by the changes in morphology and an increase in surface characterization parameters. The modification of biomolecules induced the same *I*_*D*_–*V*_*G*_ curve shift trend in the condition applied without nanomaterial (Figure S11A, Supporting Information) and with ReS_2_ (Figure S11B, Supporting Information). The most decrease in *V*_*t*_ value measured on Au‐ReS_2_‐functionalized electrodes at target detection demonstrated the effective implementation of the biosensing signal enhancement strategy (Figure S11C, Supporting Information). In the functionalization of Au‐ReS_2_ and the aptamer, conductivity changes of the sensing membrane revealed a baseline drift beyond the 95% confidence interval starting from the 8th and 6th day, respectively (Figure 3G). These results indicate that the constructed biosensing structure remains stable for up to 5 days, enabling its potential for subsequent target detection.

### 2.3 Evaluation of the In Vitro Biosensing Performance

It is essential to define the background interference signals caused by sample conditions to ensure reliable target detection.^[^ ^58^ ^]^ The biosensing cut‐off value was calculated using Equation (2).^[^ ^59^ ^]^

(2) $$\text{Biosensing}\;\text{cut−off}\;\text{value}=\Delta V_{t,\text{control}}\times 3$$

*V*_*t*,control_ represents the *V*_*t*_ value generated by the dilution conditions of the measurement samples. The biosensing cut‐off values for GzmB and PRF detection in the human serum were calculated as 0.04688 and 0.05883, respectively (Figure S12, Supporting Information). To preliminarily evaluate the practical applicability of the fabricated biosensor, Δ*V*_*t*_ was calculated using Equation (3).

(3) $$\Delta V_{t}=\frac{V_{t,\text{blank}}-V_{t,\text{target}}}{V_{t,\text{blank}}}$$

*V*_*t,blank*_ and *V*_*t,target*_ represent the *V*_*t*_ values of the blank sample and the target detection, respectively. Based on a linear regression curve consisting of Δ*V*_*t*_ for detection at each target concentration, the limit of detection (LOD) was calculated using Equation (4).^[^ ^60^ ^]^

(4) $$\text{LOD}=3.3\frac{\text{SD}}{\text{slope}}$$

**Figure 4:**
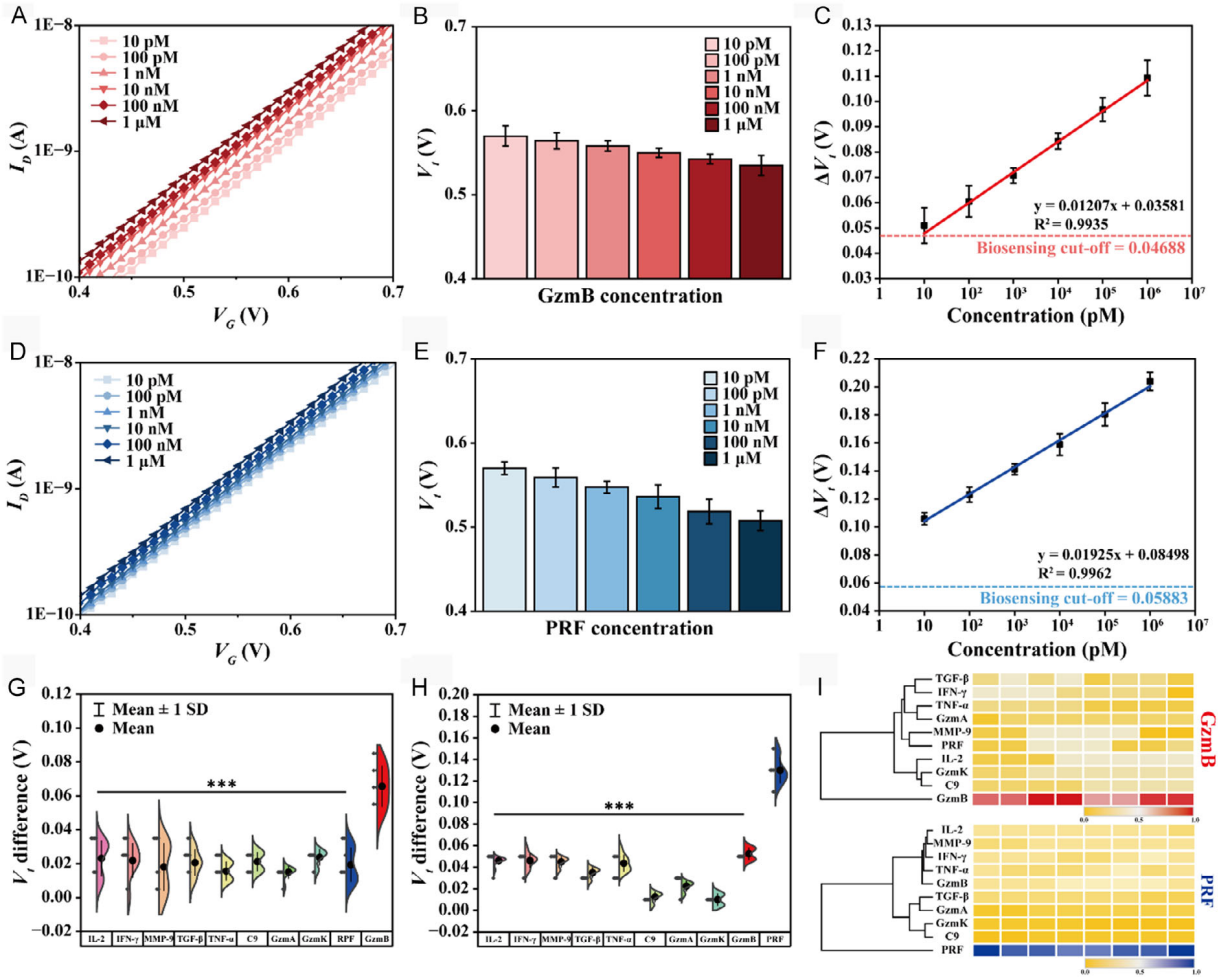
A) *I*_*D*_–*V*_*G*_ curves, B) *V*_*t*_ values, and C) calibration curve at the detection range of 10 pM to 1 μM for GzmB diluted in 10% human serum. D) *I*_*D*_–*V*_*G*_ curves, E) *V*_*t*_ values, and F) calibration curve at the detection range of 10 pM to 1 μM for PRF diluted in 10% human serum. G) Half violin plot visualization for the selectivity test of (G) GzmB and H) PRF. I) Heatmap analysis using the independent value of the selectivity test. In vitro biosensing performance evaluations were repeated to ensure reproducibility and reliability (*n* = 8). Significant differences of *V*_*t*_ difference between targets and nontargets are determined by one‐way ANOVA and were highlighted as *** *p* < 0.001.

SD is the standard deviation of the blank sample, and the slope is derived from the calibration curve. As the GzmB concentration increased, the *I*_*D*_–*V*_*G*_ curve shifted in the negative direction (**Figure** **4**A) with the decrease of *V*_*t*_ (Figure 4B). The Δ*V*_*t*_ values were linearly proportional to the log concentration of GzmB, and the LOD was calculated to be 330 fM (Figure 4C). Similarly, the negatively shifting of the *I*_*D*_–*V*_*G*_ curve was exhibited with the increasing PRF concentration (Figure 4D). This trend was confirmed by the gradual decrease in the *V*_*t*_ values (Figure 4E), showing 440 fM of LOD for PRF (Figure 4F). Respiratory diseases involve the expression of various inflammatory factors for the immune response.^[^ ^61^, ^62^ ^]^ The selectivity test was evaluated by applying the nontarget inflammatory proteins, including interleukin‐2 (IL‐2), interferon‐gamma (IFN‐γ), transforming growth factor‐β (TGF‐β), tumor necrosis factor‐α (TNF‐α), and matrix metalloproteinase‐9 (MMP‐9). In addition to reflecting clinical settings, the selective biosensing of aptamer was evaluated using homologous proteins GzmA, GzmK, and complement component 9 (C9). Nonspecific proteins exhibited significantly different detection signals (*p* < 0.001) compared with GzmB (Figure 4G) and PRF (Figure 4H), demonstrating the superior selectivity and target discrimination accuracy of the platform. Cross‐validation between the two selected biomarkers demonstrated that each target was specifically detected without interference. Heatmap analysis based on independent experimental results for selectivity evaluation showed reproducibility and intuitive selectivity (Figure 4I).

### 2.4 Evaluation of the Applicability of Respiratory Diseases in a Clinical Setting

**Figure 5:**
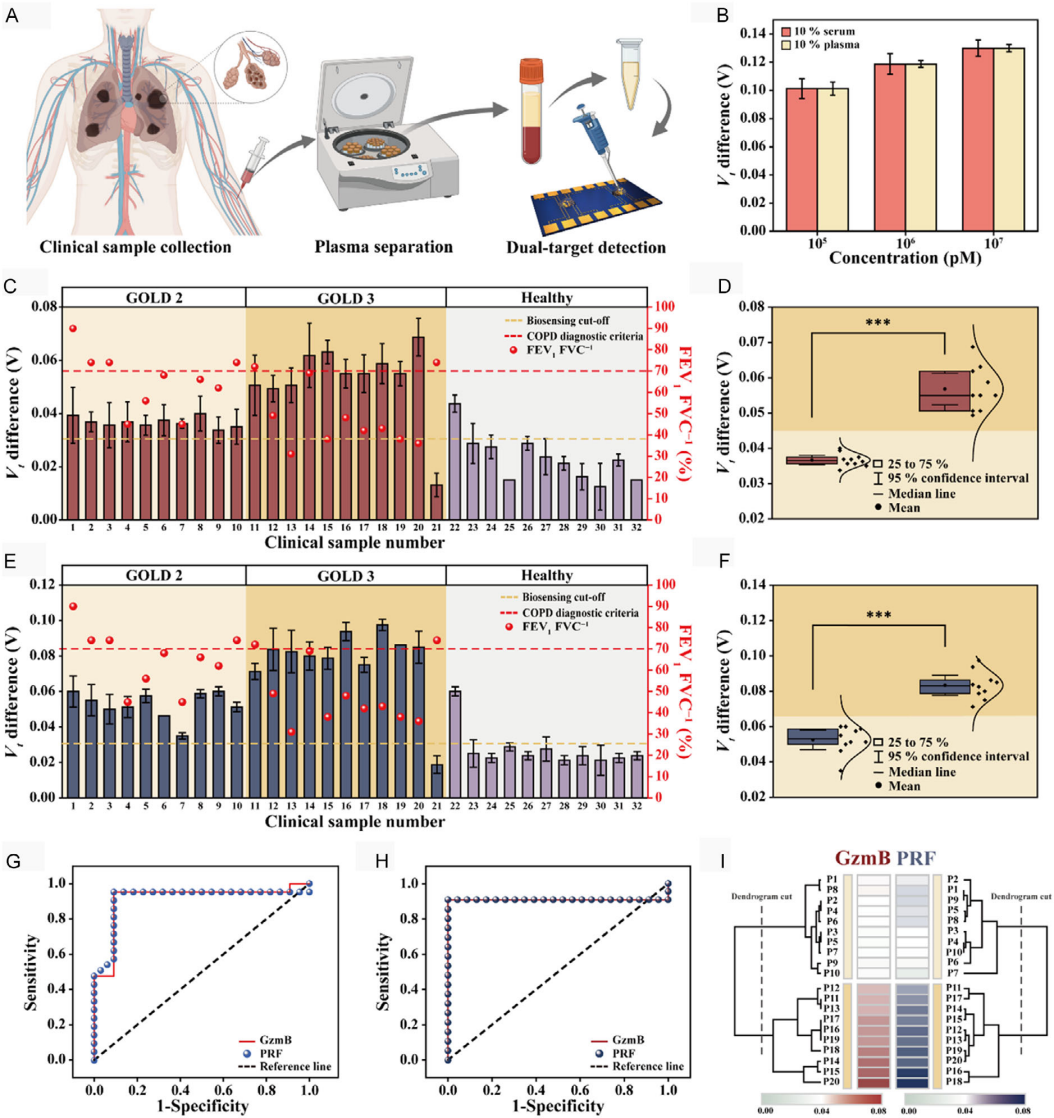
A) Schematic illustration of biosensing using a clinical sample. B) *V*_*t*_ difference measurements for the same concentration of the target diluted in 10% and 10% plasma. C) Biosensing results of GzmB in COPD patients and healthy individuals. D) Electrical measurement signals of GzmB according to the GOLD stage of bronchial dysfunction in COPD patients. E) Biosensing results of PRF in COPD patients and healthy individuals. F) Electrical measurement signals of PRF according to the GOLD stage of bronchial dysfunction in COPD patients. G) ROC curve for COPD diagnosis between COPD patients and healthy controls using GzmB and PRF detection. The optimal cut‐off values of *V*_*t*_ difference for GzmB and PRF in COPD diagnosis were 0.03125 and 0.03188, respectively. H) ROC curve for GOLD classification of COPD‐positive patients using GzmB and PRF detection. The optimal cut‐off values of *V*_*t*_ difference for GzmB and PRF in GOLD classification were 0.04469 and 0.06562, respectively. I) Heatmap analysis using GzmB and PRF biosensing results reflecting GOLD classification in COPD diagnostic sample. The target detections using clinical samples were repeated to ensure reproducibility and reliability (*n* = 8). Significant differences of *V*_*t*_ between GOLD 2 and GOLD 3 are determined by one‐way ANOVA and were highlighted as *** *p* < 0.001. Illustrations in (A) were created with BioRender.com and used with permission.

The practical clinical applicability of the biosensor was evaluated using plasma samples from COPD patients (**Figure** **5**A). According to the 2024 World Health Organization report, COPD was ranked as the 4th leading cause of global death and was predicted to become the primary contributor to mortality.^[^ ^1^, ^63^ ^]^ Given that this disease can pose a silent threat to anyone in daily life due to exposure to air pollution, biomass, and smoking in modern industrialized societies,^[^ ^63^ ^]^ it was selected to evaluate the potential of the developed biosensor for broad‐spectrum diagnosis of respiratory diseases. The biosensing cut‐off value was redefined to consider the potential noise in clinical samples (Figure S13, Supporting Information). In addition, in vitro biosensing evaluations were partially re‐performed in a plasma environment, confirming that performance was maintained in clinical sample conditions (Figure 5B). COPD treatment relies on symptom relief through bronchodilation and functional maintenance and has limited effectiveness in fundamentally restoring airway structure.^[^ ^64^ ^]^ The forced expiratory volume in 1 s (FEV_1_) and the ratio between FEV_1_ to forced vital capacity (FVC) are used for the severity classification according to Global Initiative for Chronic Obstructive Lung Disease (GOLD) and diagnosis of COPD, respectively.^[^ ^1^ ^]^ FVC can be influenced by age and sex, leading to variability in the diagnostic criteria for COPD. Even under these considerations, a subset of patients was not classified as COPD, and several among them exhibited severely impaired bronchial function (Table S5, Supporting Information). This discrepancy was likely due to interference from complications, suggesting that spirometry had limitations in accurately assessing the prognosis of COPD. The expression of inflammatory markers such as IL‐6, IL‐8, and TNF‐α was unrelated to localized functional impairments in patients with COPD.^[^ ^5^ ^]^ In contrast, increased expression of GzmB and PRF has been reported to reflect pulmonary tissue damage.^[^ ^65^ ^]^ Electrical measurements of GzmB were unaligned with conventional COPD criteria (Figure 5C), but showed a significant distribution (*p* < 0.001) that differentiated GOLD 2 from GOLD 3 patients (Figure 5D). Similar detection trends were observed in PRF measurements (Figure 5E), which are indicators of the GOLD classification levels of COPD patients (*p* < 0.001) (Figure 5F).

Dual biomarker detection via biosensor achieved an area under the curve (AUC) of 0.913, a sensitivity of 95.2%, and a specificity of 90.9% in COPD diagnosis (Figure 5G). There are concerns regarding the reliability of classifying COPD severity based solely on FEV_1_ values of individual patients.^[^ ^1^ ^]^ Nevertheless, reduced FEV_1_ in patients with airflow obstruction has been associated with increased mortality,^[^ ^66^ ^]^ indicating that it serves as a major determinant of bronchial functional status and inflammatory burden. GzmB and PRF identified GOLD severity with an AUC of 0.909, a sensitivity of 90.1%, and a specificity of 100.0% (Figure 5H). The GzmB and PRF detection results of the GOLD 2 and GOLD 3 sample groups were perfectly separated by dendrogram cut (Figure 5I), demonstrating that the proposed biomarkers can reflect the GOLD classification. Previous reports have shown that measured FEV_1_ in COPD patients has a significant positive correlation with GzmB‐expressing cells,^[^ ^19^ ^]^ relevant to the GzmB biosensing results of the discussed FET‐type aptasensor. The proposed dual‐respiratory disease biomarker biosensing platform can serve as a criterion for determining whether manifestations of respiratory diseases associated with GzmB activity depend on PRF. For example, independent expression of GzmB under respiratory syncytial virus infection may reflect modulation of immune pathology during acute infection.^[^ ^67^ ^]^ The co‐expression of GzmB and PRF in respiratory diseases suggests their co‐involvement in the pathological progression, thereby supporting their potential use as biomarkers to identify respiratory conditions associated with cytotoxic immune activation. The clinical samples used in this study were derived from plasma; therefore, there are inherent limitations in capturing in vivo alterations arising from localized pathophysiological processes in the lungs or bronchi. Nevertheless, given the strong association between GzmB and PRF with bronchial dysfunction, the introduced platform has potential as a clinically relevant tool for diagnosing and monitoring bronchial diseases. Thus, validation of the expression patterns of GzmB and PRF in various respiratory diseases and clinical samples will advance the proposed biosensing system for enabling the practical application in developing a respiratory disease assessment platform.

In summary, the developed device offers a potential diagnostic technique capable of reflecting bronchial function and the degree of airway damage in respiratory diseases through electrical biosensing of GzmB and PRF. This not only provides a distinct advantage in detecting physiological changes in patients whose respiratory impairments remain undetected using spirometry but also provides superior detection times and LODs compared to existing respiratory disease biosensing platforms (**Table** **1**). In addition, the sensor showed stable detection signals within the 95% confidence interval for 5 days at 4 °C (Figure S14A, Supporting Information) and 25 °C (Figure S14B, Supporting Information) with an inter‐chip coefficient of variation (CV) of less than 10% (Table S6, Supporting Information). These results suggest that the sensor can provide reliable and reproducible short‐term diagnoses of respiratory diseases based on GzmB and PRF. Since the gate of the fabricated EG‐FET biosensor is replaced with an electrode, the entire device performance is unaffected by contamination or damage to biological samples.^[^ ^68^ ^]^ This modular structure ensures both efficiency and economy by minimizing measurement interference and enabling platform reusability through easy membrane replacement.^[^ ^69^ ^]^ The used sensing membrane showed a stable operational response in the measurement signal during repeated target detection and washing up to the 5^th^ cycle (Figure S15, Supporting Information). This indicates robust reusability without the need to replace even the sensing membrane and demonstrates performance comparable to that of recent FET biosensing platforms (**Table** **2**).

**Table 1:** Comparison of biosensing performance with previous respiratory disease diagnostic biosensors. NM means not mentioned.

Probe	Target protein	Disease	Detection method	Sample condition	Detection time	Detection range	LOD	REF
Peptide	Hemagglutinin (HA) proteins of H5N1/H1N1	Influenza virus	Differential pulse voltammetry	1X phosphate‐buffered saline (PBS) buffer and artificial human blood plasma (ABP)	360 min	25.00 to 300.00 nM	PBS: 2.29 nM for H5N1/3.09 nM for H1N1 ABP: 2.39 nM for H5N1/3.63 nM for H1N1	[76]
Peptide	HA proteins of H5N1	Influenza virus	Square wave voltammetry (SWV)	Spiked human plasma	30 to 60 min	31.20 to 500.00 nM	3.26 nM	[77]
Antibody	CFP10‐ESAT6 protein	Tuberculosis (TB)	EIS	1X PBS buffer	240 min	7.58 to 758.00 pM	72.70 pM	[78]
Antibody	Heat shock protein 16.3	TB	SWV	Spiked human serum	20 min	0.49 pM to 1.49 nM	490 fM	[79]
Aptamer	IFN‐γ	TB	Chronocoulometry	0.1 M PBS	NM	50.00 fM to 1.00 pM	405 fM	[80]
Antibody	SARS‐CoV‐2 spike antigen protein	SARS‐CoV‐2	FET	0.01X PBS buffer	NM	166.66 pM to 66.66 μM	166.66 pM	[81]
Epitope	MMP‐1	Idiopathic pulmonary fibrosis	EG‐FET	10 mM MES buffer	60 min	10.00 to 250.00 nM	100 pM	[82]
Aptamer	GzmB/PRF protein	COPD	EG‐FET	10% human serum	10 min	10.00 pM to 1.00 μM	330/440 fM	This study

**Table 2:** Comparison of the developed biosensing platform with existing FET‐type biosensors. NM means not mentioned.

FET‐type	Signal amplification strategy	Target detection stability	Reusability	Detection time	LOD	REF
Back gate	Multi‐walled carbon nanotubes (MWCNT)/MXene/MoS_2_	16 days	4 cycles	NM	500.00 pM	[83]
Buried gate	MWCNT	14 days	NM	8 min	150.00 pM	[84]
Double gate	Graphite	NM	NM	NM	13.30 nM	[85]
Liquid gate	Reduced graphene oxide	15 days	4 cycles	120 min	1.00 fM	[86]
Liquid gate	Camphor–rosin clean transfer‐based graphene	12 h	NM	NM	100.00 fM	[87]
Liquid gate	SiO_x/_SiN_x/_InN	NM	NM	30 min	2.50 pM	[88]
Liquid gate	Argon etching‐based MoS_2_	NM	NM	10 min	60.00 fM	[89]
Extended gate	MXene	6 days	NM	NM	10.64 pM	[49]
Extended gate	UiO‐66	NM	NM	10 min	36.20 fM	[90]
Extended gate	Indirect assay format	NM	NM	15 min	28.00 pM	[91]
Extended gate	Extended gate structure	NM	NM	NM	2.90 μM	[92]
Extended gate	Au‐ReS_2_	5 days	5 cycles	10 min	330.00/440.00 fM	This study

## 3 Conclusion

In this study, a novel EG‐FET‐based platform was developed for the simultaneous detection of GzmB and PRF, aiming to overcome the limitations of spirometry‐based diagnosis in respiratory diseases. To enable robust detection in clinical settings, the synthesized aptamers were truncated to enhance their binding affinities for the target molecules. The applied strategies improved both sensitivity and response time, and the system performed successfully even in human serum, indicating its potential for clinical use. The ability of the developed device to appropriately reflect the bronchial function status in actual patients with COPD provides clear evidence that the entire process—from the selection of target biomarkers designed to evaluate localized immune responses in respiratory diseases to the construction of a biosensing system—was successfully aligned with its intended purpose. The technological sophistication and clinical applicability of the proposed sensor suggest its potential not only for assessing bronchial function but also for early diagnosis and therapeutic monitoring of respiratory diseases. Therefore, the proposed platform holds promise as a potential alternative to conventional spirometry and may represent a milestone in the establishment of frontline diagnostic strategies for the proactive detection of respiratory diseases.

## 4 Experimental Section

4.1

4.1.1

##### Materials

Recombinant Human GzmA, GzmB, GzmK, IFN‐γ, IL‐2, MMP‐9, TNF‐α, and TGF‐β proteins were purchased from Sino Biological (Beijing, China). Recombinant Human C9 protein was purchased from Abcam (Cambridge, UK). Recombinant Human PRF protein was purchased from RayBiotech (GA, USA). Bis‐dPEG_5_‐NHS ester was purchased from Vector Laboratories (Newark, CA, USA). Pooled human plasma apheresis derived was purchased from Innovative Research (Novi, MI, USA). PCR master mix and 100 bp DNA size marker for electrophoresis were purchased from Bioneer (Daejeon, South Korea). The streptavidin magnetic beads were purchased from GenScript (NJ, USA). Sulfo‐NHS‐LC‐Biotin was purchased from Thermo Fisher Scientific (MA, USA). The XELEX DNA Core kit with the DNA library with 5′‐TGA CAC CGT ACC TGC TCT‐N40‐AAG CAC GCC AGG GAC TAT‐3′ was purchased from EURx (Gdansk, Poland). (3‐aminopropyl)triethoxysilane (APTES), ammonium perrhenate (NH_4_ReO_4_), bovine serum albumin (BSA), hydroxylamine hydrochloride (NH_2_OH·HCl), thioacetamide (TAA), gold(III) chloride solution (HAuCl_4_), polyvinyl pyrrolidone (PVP), sodium borohydride (NaBH_4_), human serum, and cysteamine were purchased from Sigma–Aldrich (St. Louis, MO, USA). The n‐type silicon wafer was manufactured by MCL Electronics Materials (Luoyang, China). The Au micro‐gap electrode was manufactured by SNI Technology (Seoul, South Korea). 4‐(2‐Hydroxyethyl)‐1‐piperazineethanesulfonic acid (HEPES), acetone (ACE; C_3_H_6_O), ethyl alcohol (EtOH; C_2_H_5_OH), and sodium citrate anhydrous (C_6_H_5_Na_3_O_7_) were purchased from Daejung (Gyeonggi‐do, South Korea). GzmB and PRF aptamers modified with fluorescein phosphoramidite (FAM), amine, and thiol groups were synthesized from Bionics (Seoul, South Korea).

##### SELEX Process and Aptamer Selection

The proteins at 0.25 mg mL^−1^ were biotinylated by reacting with 2 mM Sulfo‐NHS‐LC‐Biotin at 4 °C for 1 h. The unreacted substances were transferred to a 3000 NMWL Amicon filter unit (Sigma–Aldrich) and removed by centrifugation at 2400 × g for 20 min. The filtrate was incubated with 100 μL of streptavidin magnetic beads at 4 °C for 1 h to form magnetic bead@protein complexes, and the residues were washed with 500 μL of 1X SELEX buffer. Following mixing with 20 μL of random DNA library and 180 μL of 1X SELEX buffer, the complex was incubated at 4 °C for 1 h. Unbound DNA was removed by washing with 200 μL of 1X SELEX buffer, and DNA binding to the target protein was recovered by heating at 85 °C for 10 min using a heat block (BioTek, VT, USA). Negative SELEX, which is known as a strategy to increase the target protein selectivity of the aptamer,^[^ ^70^ ^]^ was performed in the same manner as above using BSA as a nontarget protein, but nucleic acids that were unbound to magnetic bead@protein were harvested to conduct subsequent SELEX rounds. Subsequently, PCR was performed on the aptamer candidates using an iCycler (Bio‐Rad, CA, USA) and the above procedures were repeated until aptamers with suitable yield were obtained. The PCR conditions were as follows: Initial denaturation at 95 °C for 2 min; 13 cycles at 95 °C for 30 s, 55 °C for 1 min, 72 °C for 3 min; Final extension at 70 °C for 5 min. The final round SELEX product was sequenced by Solgent (Seoul, South Korea). These were analyzed for their two‐dimensional (2D) structures and Δ*G* using the mFold application (https://www.unafold.org/). The binding affinity of the selected candidates was evaluated by modifying the FAM dye at the 5′ end of aptamers. Fluorescence measurements were performed using a Synergy LX Multimode Reader (BioTek) with absorption and emission wavelengths at 480 and 528 nm, respectively.

##### Aptamer Truncation and Target Docking Simulation

The primer‐binding regions were truncated stepwise while preserving the stem‐loop structure of the aptamer. The three‐dimensional structures of GzmB (PDB ID: 1FQ3) and PRF (UniProt ID: P14222) were obtained from the AlphaFold Protein Structure Database (https://alphafold.ebi.ac.UK/) and RCSB PDB database (https://www.rcsb.org/), respectively. The protein PDB files and aptamer sequences were uploaded to the HDOCK server (https://hdock.phys.hust.edu.cn/), and all parameters were set to default for the docking simulation. The docking model with the highest docking score was analyzed for noncovalent interactions using the Protein–Ligand Interaction Profiler web tool (https://www.plip‐tool.biotec.tu‐dresden.de/plip‐web/plip/index/) and was visualized using PyMOL molecular visualization software (Version 3.1.3; Schrödinger, LLC., New York, NY, USA). Among the constructed models, the model with the highest confidence was selected, and generally, a confidence score higher than 0.7 indicates a high probability of the binding event occurring. A low docking score indicates an increased likelihood of binding, and ligand RMSD values are used to assess the structural stability of molecules in molecular dynamics simulations.^[^ ^71^ ^]^

##### Circular Dichroism (CD) Spectroscopy

CD analysis was conducted using a Chirascan spectropolarimeter (Applied Photophysics, Leatherhead, UK) with a photomultiplier tube detector. Aptamers were prepared at 0.5 mg mL^−1^ in 10 mM potassium phosphate buffer (pH 7.4) and measured from 180–280 nm with a bandwidth of 1 nm, data pitch of 1 nm, scan speed of 240 nm min^−1^, and response time of 0.25 s per point. Data were presented as molar ellipticity in millidegrees (mdeg).

##### DP‐SIS Measurement

The n‐type silicon wafers were sequentially washed with ACE and EtOH to eliminate organic contaminants, followed by N_2_ gas drying between each step. To achieve a clean and hydroxyl‐rich surface, the wafers were subsequently immersed in freshly prepared piranha solution (H_2_SO_4_:H_2_O_2_  = 3:1, v/v). The cleaned substrates were then functionalized by incubating them in a 2% solution of APTES for 12 h at ambient temperature. After silanization, excess reagent was removed through thorough rinsing with anhydrous ethanol, and the wafers were cured at 70 °C for 30 min. The treated silicon chips were then integrated into a dual‐prism configuration to assemble the DP‐SIS sensing platform. For aptamer immobilization, NH_2_‐terminated surfaces were activated using NHS‐ester chemistry to enable covalent binding of 5 μM of aptamers. Following immobilization, unreacted functional groups were passivated with 0.5% BSA prepared in PBS. All surface‐modification procedures were performed under a controlled flow rate of 30 μL min^−1^. Target protein samples were prepared in PBS containing 0.05% sodium dodecyl sulfate, serially diluted on an exponential scale to enable direct detection. The real‐time binding interactions were monitored at a constant flow rate of 100 μL min^−1^.

##### Au‐ReS_*2*_ *Synthesis and Characterization*

ReS_2_ and Au‐ReS_2_ were synthesized based on previous studies with slight modifications.^[^ ^72^, ^73^ ^]^ Ammonium perrhenate of 40 mg and TAA of 60 mg were completely dissolved in 30 mL of deionized water (DIW). The solution was reacted in a hydrothermal synthesis reactor at 200 °C for 24 h. The mixture was centrifuged at 1070 × g for 30 min at room temperature. After removing the supernatant, the pellet was washed with DIW. The washed pellet underwent the centrifugation procedure, was dispersed in 70% EtOH, and dried at 75 °C to yield ReS_2_ NPs. For Au‐ReS_2_ synthesis, 3% gold(III) chloride solution and 5 mg PVP were mixed into a 0.4 mg mL^−1^ ReS_2_ colloidal solution dispersed in DIW. Thereafter, 50 mM sodium citrate anhydrous and 100 mM sodium borohydride were added to the mixture. After stirring the solution for 24 h, Au‐ReS_2_ powder was obtained through the washing and isolation procedures. The optical properties of ReS_2_ and Au‐ReS_2_ were analyzed using a ultraviolet‐visible‐near infrared (UV‐vis‐NIR) spectrometer (UV‐1800, SHIMADZU, Kyoto, Japan). The synthesized NPs were characterized using field‐emission SEM (JSM‐7100 F, JEOL, Tokyo, Japan), transmission electron microscopy (TEM; JEM‐2100 F, JEOL, Tokyo, Japan), and energy‐dispersive X‐ray spectroscopy (EDS) analysis. The X‐ray diffraction (XRD; Empyrean, Malvern Panalytical, Malvern, UK) analysis using Cu Kα (λ = 1.5406) in the 2*θ* range of 5 to 90° and XPS (PHI 5000 Versa Probe III, ULVAC‐PHI, Kanagawa, Japan) analyses using Al Kα (1486.6 eV) source and C 1 s peak (284.8 eV) for calibration were also conducted to confirm the successful synthesis of Au‐ReS_2_.

##### Electrode Functionalization for Biosensing

The Au electrode was sonicated using 99.5% ACE solution for 5 min to remove the surface impurities. The cleaned Au electrode was sequentially washed with EtOH and DIW, and dried using N_2_ gas. To activate the amine groups on the sensing membrane, 10 mM cysteamine was incubated for 1 h. Subsequently, 5  μL of the Au‐ReS_2_ solution was drop‐cast onto the electrode surface and incubated for 1 h. The thiol‐modified truncated aptamer was immobilized onto the Au‐ReS_2_ surface by incubation for 3 h. Target binding events were accelerated by applying ACEF using a function generator (Tektronix, Beaverton, OR, USA) with a 3 V amplitude of AC at 1 MHz frequency for 10 min.^[^ ^27^ ^]^ After each functionalization step, the sensing membrane was washed with DIW and dried using N_2_ gas. The biosensing structure constructions were characterized using atomic force microscopy (AFM; XE7, Park Systems, Suwon, South Korea). A noncontact cantilever (PPP‐NCHR, Park Systems) was used to prevent the damage to biomolecules.^[^ ^28^ ^]^ The resonance frequency and spring constant were set to 230 to 305 kHz and 20 to 30 N m^−1^, respectively, and other measurement parameters were optimized automatically.^[^ ^74^ ^]^

##### EG‐FET Biosensing Condition

An n‐type metal‐oxide‐semiconductor FET (CD4007, Texas Instruments, TX, USA) was used for constructing the FET‐based biosensing system. The Au electrode was connected to the gate terminal of the FET converter and functioned as an EG. Electrical measurements were conducted in 1X PBS at pH 7.5 to minimize biomolecular denaturation and maintain stable ion strength for consistent signal values. While the source terminal was electrically grounded, the *V*_*G*_ was swept from 0 to 2 V using an Ag/AgCl reference electrode (CHI111, CH Instruments, Austin, TX, USA), and *I*_*D*_ was measured at 0.01 V intervals of drain voltage. Biosensing was performed using a source meter (Keithley 2614B, Keithley, CLE, USA), and the detection signal was analyzed based on the correlation between *I*_*D*_ and *V*_*G*_. The *V*_*t*_ was defined as *V*_*G*_ at which *I*_*D*_ ranged from 1 μA to 1 nA.^[^ ^49^ ^]^ EIS was performed on a workstation (CHI760E, CH Instruments) using a three‐electrode system comprising working, counter, and reference electrodes. The measurements were performed in a buffer containing 10 mM HEPES (pH 7.0) and 5 mM [Fe(CN)_6_]^3−/4−^, under a frequency range of 1 Hz to 100 kHz with an initial voltage of 0.25 V.

##### Clinical Sample Preparation

Patients with COPD were prospectively enrolled at the Yeungnam University Medical Center (Daegu, South Korea) between March 4, 2021, and December 31st, 2023. Eligibility criteria required patients to be aged ≥ 60 years and have a high symptom burden, defined as a COPD assessment test (CAT) score ≥ 10 or a modified Medical Research Council (mMRC) dyspnea scale ≥ 2, in accordance with the 2023 GOLD guidelines.^[^ ^75^ ^]^ The exclusion criteria were as follows: 1) patients with mild symptoms who did not meet the high‐risk classification based on age and clinical presentation; 2) those transferred to other institutions; and 3) those discharged prior to data collection. All participants provided written informed consent, and the study protocol (institutional review board (IRB) 2021‐02‐053) was approved by the IRB of Yeungnam University Hospital (Daegu, South Korea). Upon enrollment, clinical and demographic information, including age, sex, smoking history, comorbidities, mMRC, CAT scores, and current medications, was obtained using a questionnaire. The pulmonary function was assessed using standard spirometry. For plasma isolation, 9 mL of whole blood from all patients was collected into vacutainer tubes containing anticoagulants. Within 12 h of collection, samples were centrifuged at 2000 × g for 5 min, and supernatant plasma was stored at −80 °C.

##### Statistical Analysis

Statistical analysis was performed using SPSS software (Version 19.0; IBM Corp., Armonk, NY, USA) to calculate the standard and equilibration errors for each treatment group. Significant differences were assessed using one‐way analysis of variance, followed by Tukey and LSD algorithm for cross‐validation. Statistical significance was defined as *** *p* < 0.001.

## Supporting Information

Supporting Information is available from the Wiley Online Library or from the author.

## Conflict of Interest

The authors declare no conflict of interest.

## Supporting information

Supplementary Material

## Data Availability

The data that support the findings of this study are available in the supplementary material of this article.
