# Supplementary material for: Novel Respiratory Disease Diagnosis Tool: Development of an Au‐ReS2‐Functionalized Extended‐gate Field‐Effect Transistor‐Type Aptasensor for Simultaneous Detection of Granzyme B and Perforin
Source: Small Sci. 2026 Jan 14;6(1):e202500485. doi: 10.1002/smsc.202500485 (PMC12806331; doi:10.1002/smsc.202500485)
Supplement: Supplementary file 1 — Supplementary Material [file SMSC-6-e202500485-s001.pdf]

Supporting Information

Novel Respiratory Disease Diagnosis Tool: Development of an Au-ReS_2-functionalized Extended-gate Field-effect Transistor-type Aptasensor for Simultaneous Detection of Granzyme B and Perforin

Seokho Jung, Minyoung Ju, Hyunjun Park, Sunggu Kang, Jungbum Kim, Yoseph Seo, Jengmin Kang, Jong Geol Jang, Jung Hyun Choi, Dong Hyung Kim, Chulhwan Park, Min-Ho Lee, Wonhwa Lee*, and Taek Lee**

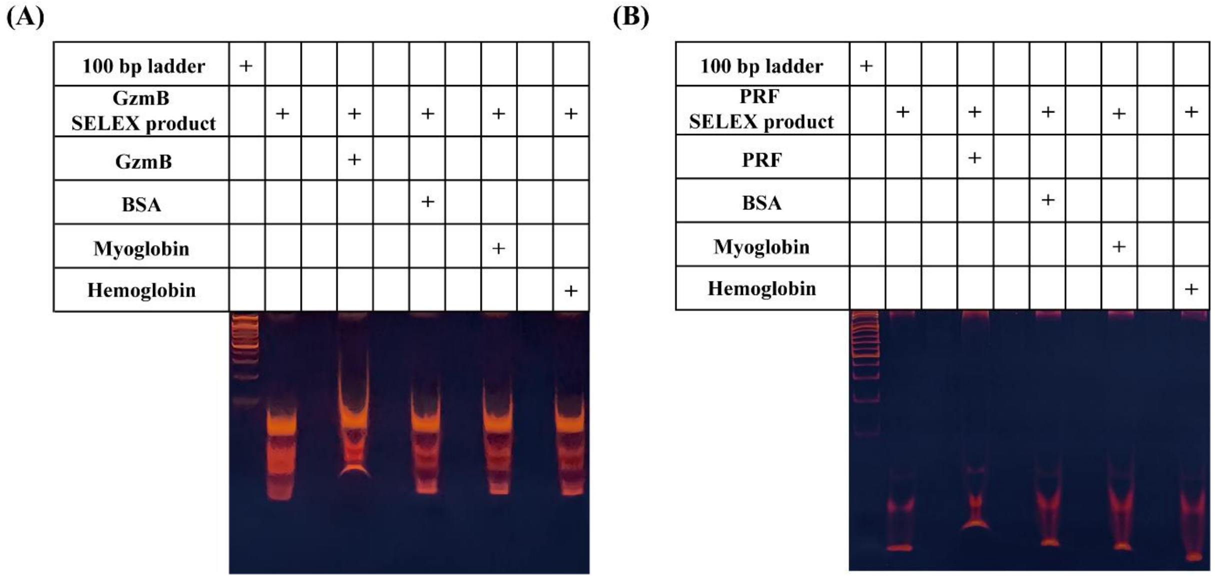

Figure S1. Binding test results of (A) GzmB and (B) PRF using SELEX product at 8 % Tris-borate-EDTA polyacrylamide gel electrophoresis. The plus marks represented the loaded reagent detail.

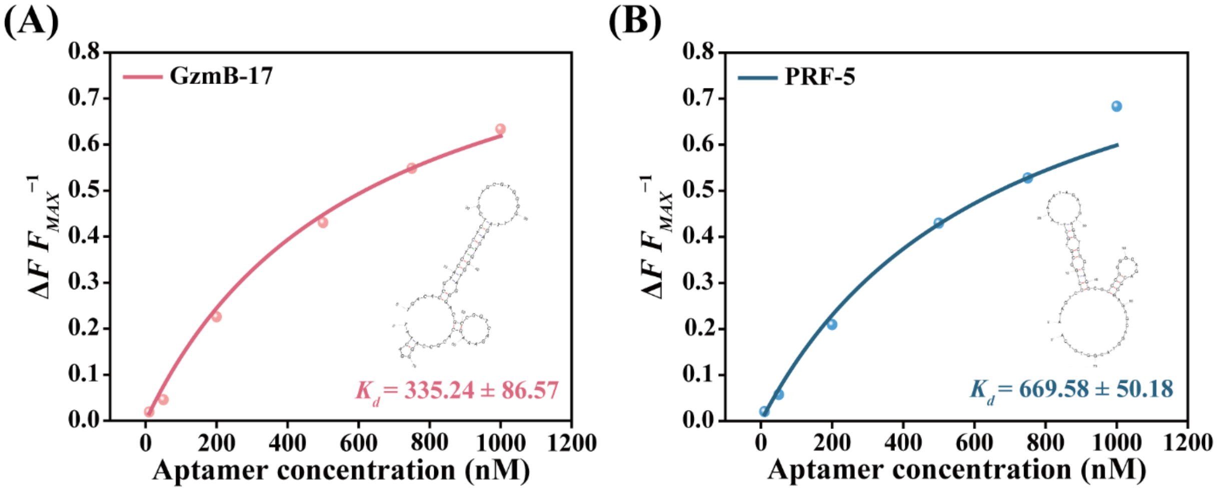

Figure S2. Binding affinity test (n = 3) results of (A) GzmB-17 and (B) PRF-5 aptamer.

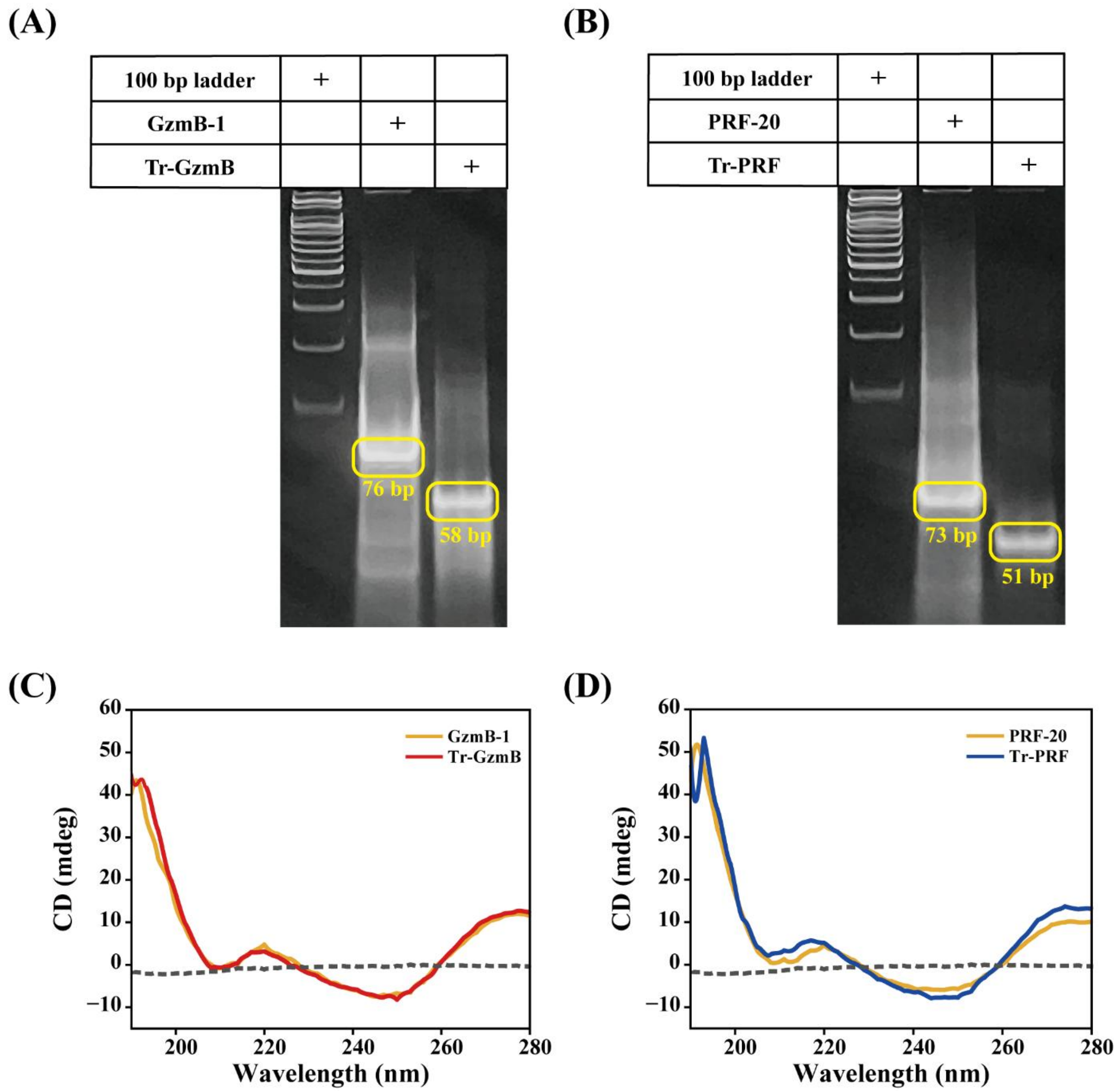

Figure S3. Gel electrophoresis results of without and with truncation process of (A) GzmB and (B) PRF. The plus marks represented the loaded reagent detail. Comparison of CD analysis results of pre- and post-truncation aptamers of (C) GzmB and (D) PRF. The gray dotted line represents the baseline according to the aptamer diluted condition.

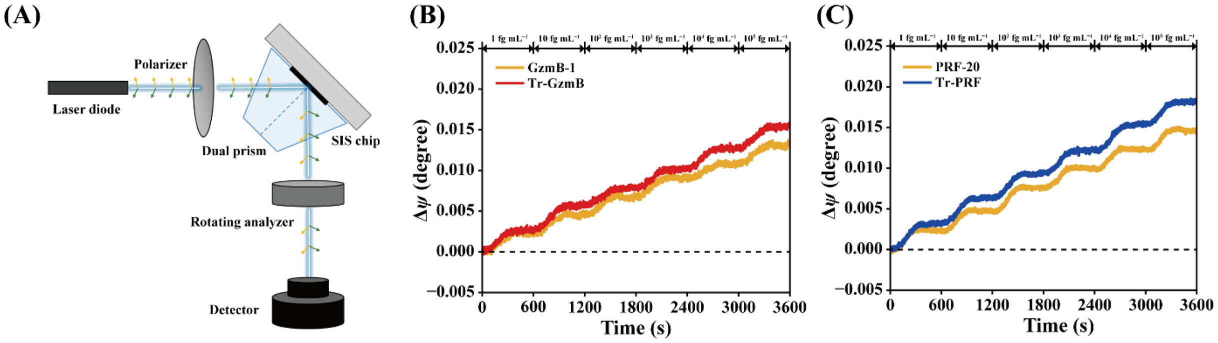

Figure S4. (A) Schematic illustration of the DP-SIS biosensing process. The real-time binding monitoring results obtained from the pre- and post-truncation (B) GzmB and (C) PRF aptamer-modified DP-SIS sensor following injection of respective targets.

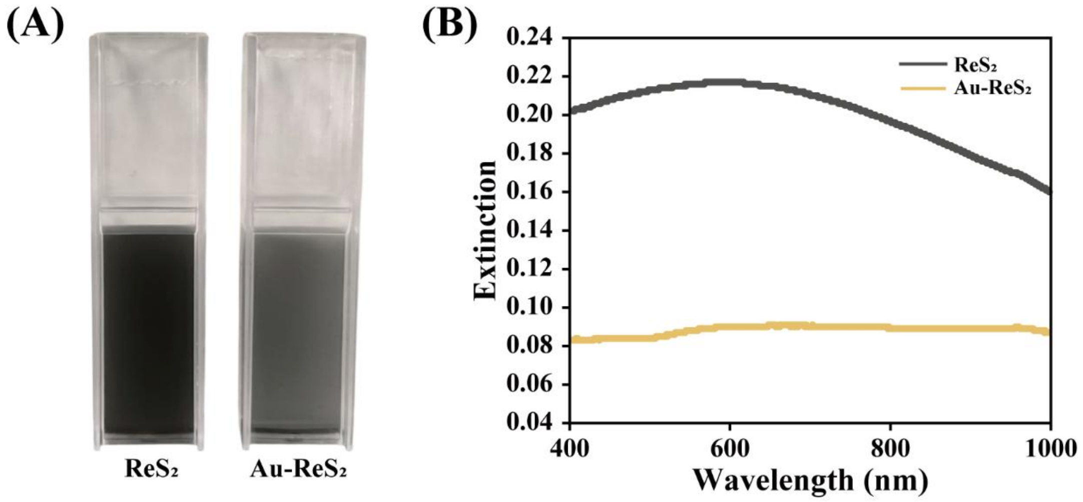

Figure S5. (A) Digital photo image of ReS_2 and Au-ReS_2 at the same concentration of 0.385 mg mL^{-1}. (B) UV-vis-NIR absorption spectra result of ReS_2 and Au-ReS_2.

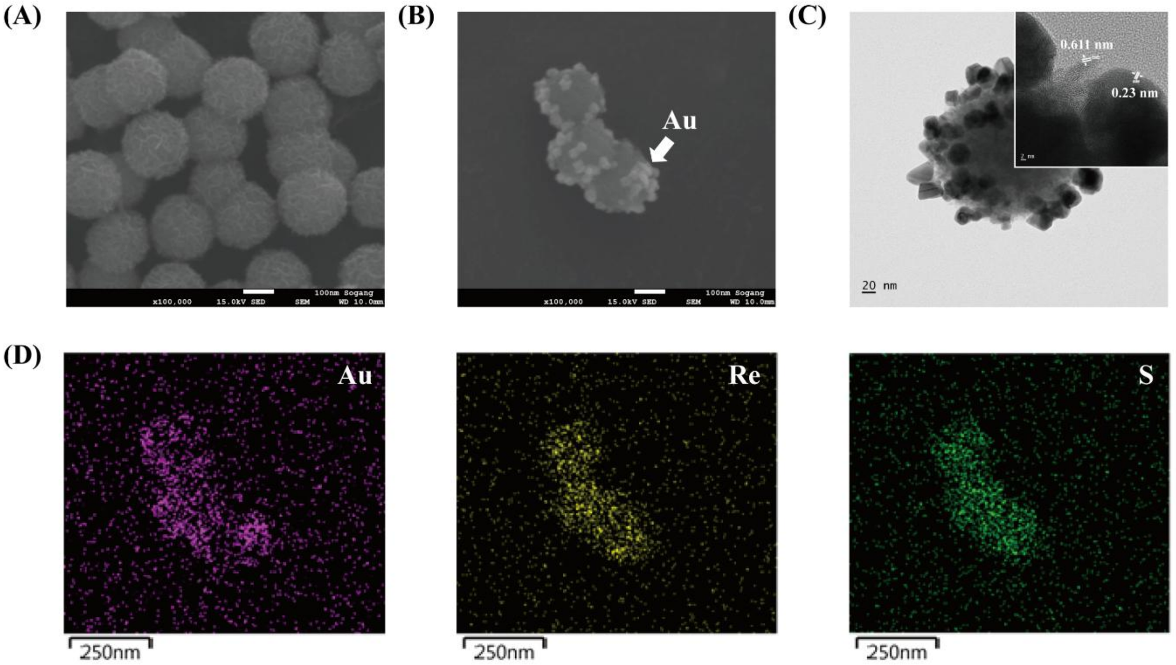

(E)

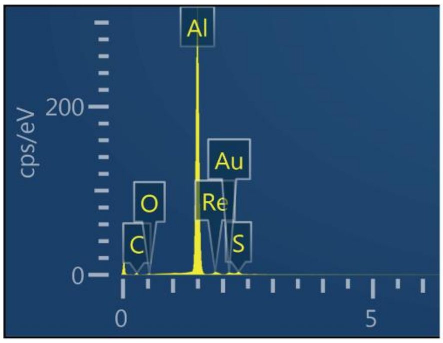

(F)

Element	Apparent concentration	Wt %	Wt % sigma	Atomic %
C	0.11	11.17	0.38	23.84
O	0.10	1.33	0.09	2.14
Al	9.61	75.13	0.41	71.38
S	0.11	1.51	0.06	1.21
Re	0.18	3.51	0.19	0.48
Au	0.41	7.35	0.24	0.96

Figure S6. SEM image analysis of (A) ReS_2 and (B) Au-ReS_2 particles. (C) TEM image, (D) SEM-EDS analysis, (E) EDS mapping image, and (F) quantitative results of EDS analysis of synthesized Au-ReS_2 particles.

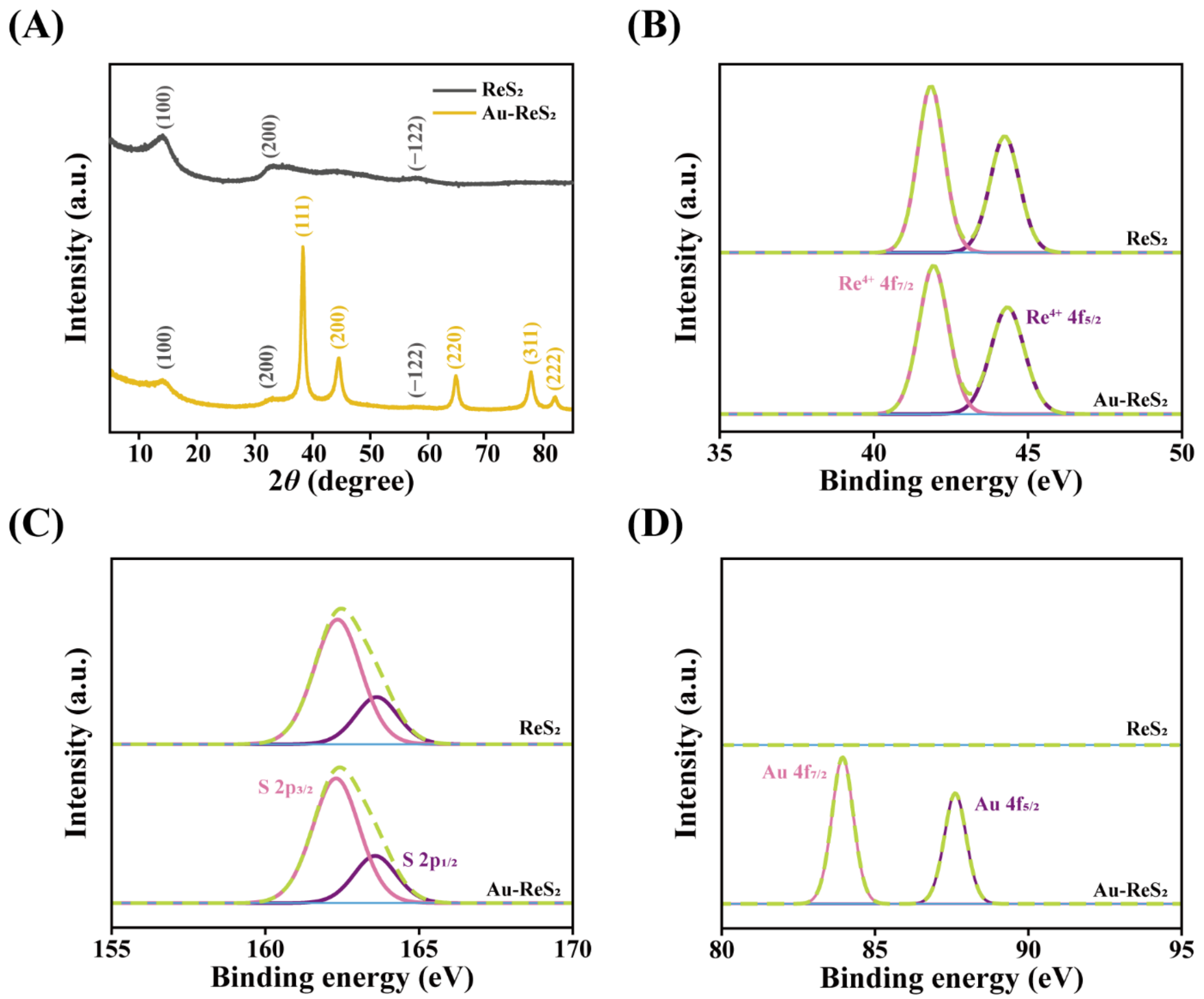

Figure S7. (A) XRD patterns for ReS_2 and Au-ReS_2. C 1s fitted XPS components of ReS_2 and Au-ReS_2 recorded in (B) Re^{4+} 4f, (C) S 2p, and (D) Au 4f.

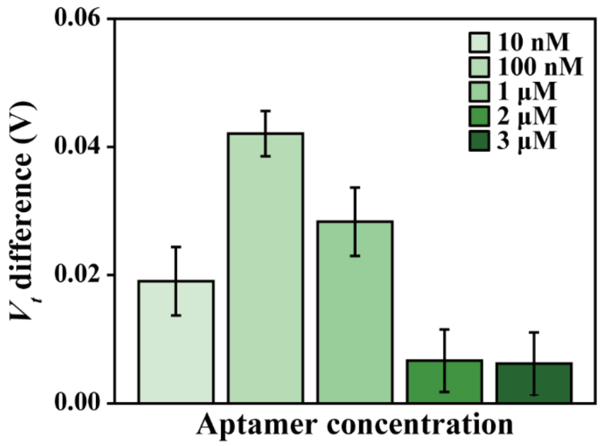

Figure S8. V_t difference values according to various aptamer concentrations (n = 8).

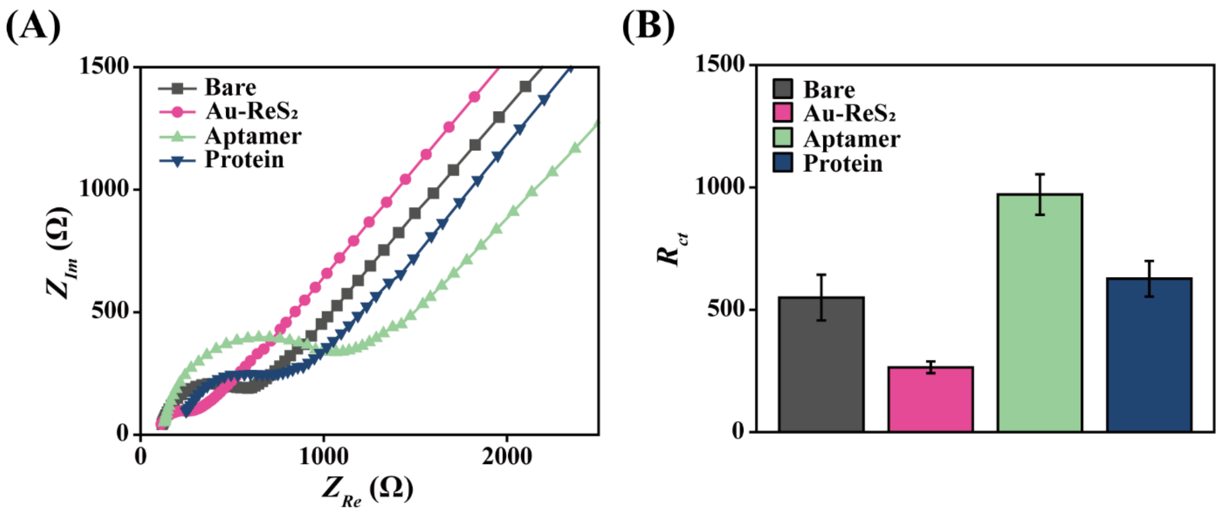

Figure S9. (A) Nyquist plots and (B) R_{ct} values of sequential sensing membrane functionalization step (n = 5).

(A)

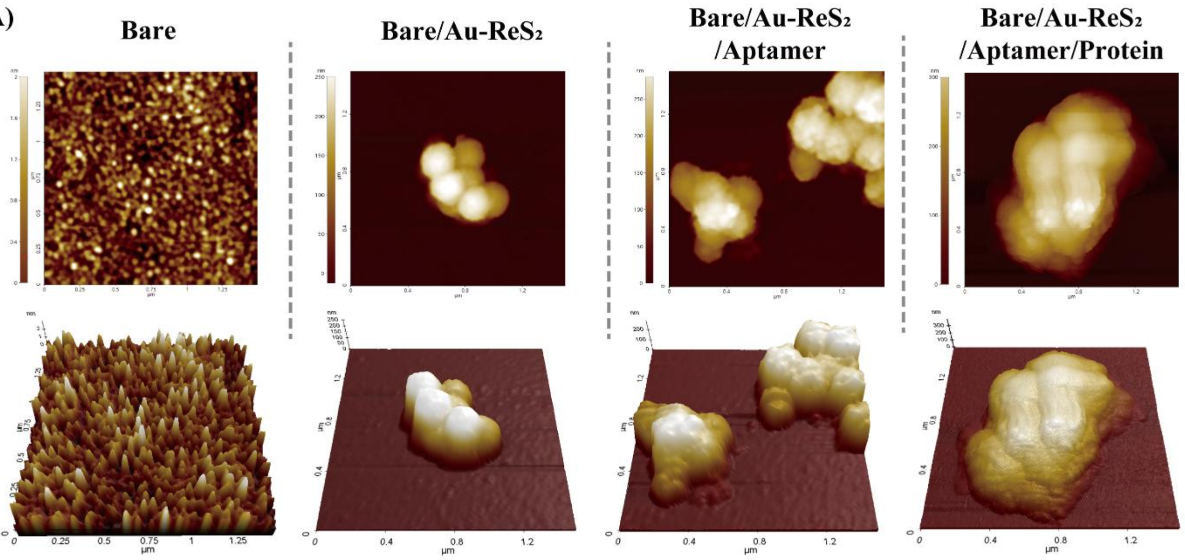

(B)

	Bare	Bare/Au-ReS_2	Bare/Au-ReS_2 /Aptamer	Bare/Au-ReS_2 /Aptamer/Protein
Vertical distance (nm)	0.678 ± 0.042	242.408 ± 1.643	258.759 ± 1.713	280.776 ± 1.259
RMS roughness (nm)	0.522 ± 0.146	41.613 ± 2.697	50.012 ± 2.242	53.130 ± 1.071
Roughness average (nm)	0.445 ± 0.130	33.116 ± 2.093	44.544 ± 1.522	46.404 ± 2.180

Figure S10. (A) AFM images and (B) surface analysis results at each functionalized electrode.

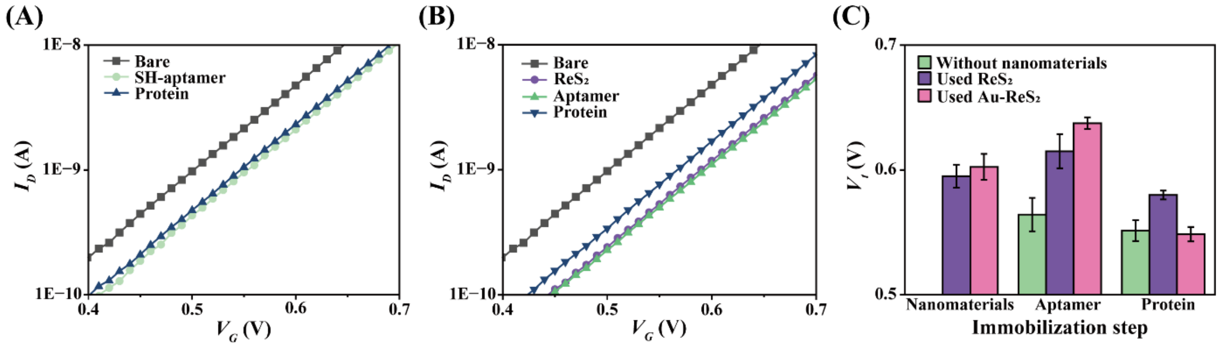

Figure S11. I_D-V_G curves of the sensing membrane applied (A) without NPs and (B) with ReS_2. (C) V_t values applied without NPs, with ReS_2, and with Au-ReS_2 at each biosensing structure construction step (n = 8).

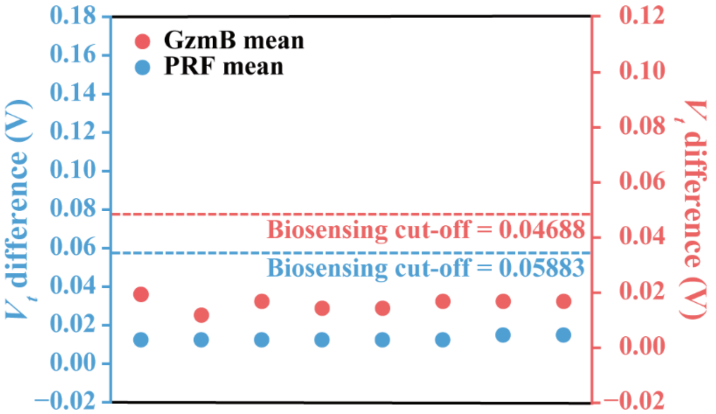

Figure S12. Biosensing cut-off values for target detection based on background signal of control (n = 8).

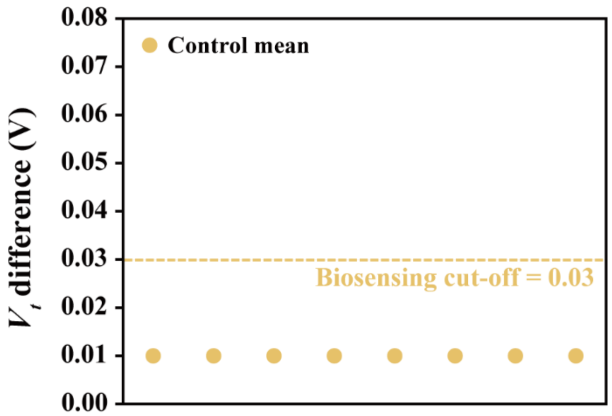

Figure S13. Biosensing cut-off value according to interference signal caused by clinical samples (n = 8).

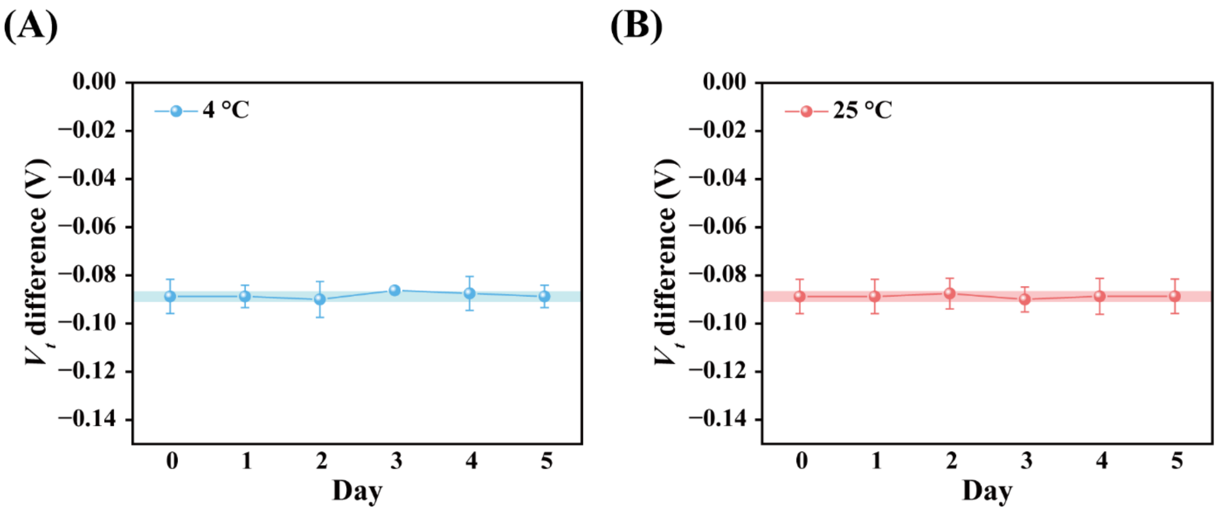

Figure S14. Target detection stability of the biosensor at (A) 4 and (B) 25 °C (n = 8). The blue and red boxes represent the 95 % confidence intervals based on the measurement signal at 0 day under 4 and 25 °C, respectively.

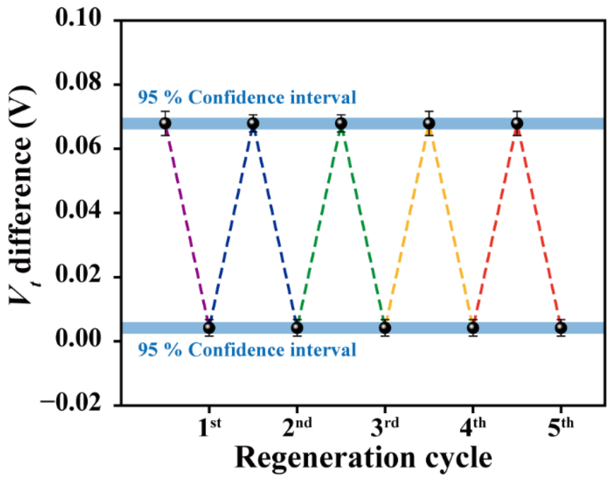

Figure S15. Reusability test of the fabricated aptasensor for target detection (n = 8).

Table S1. Sequence details of GzmB and PRF aptamers used in this study.

Protein	Aptamer	Sequences (5'-3')	ΔG value (kcal mol^{-1})
Granzyme B	GzmB-1	TGA CAC CGT ACC TGC TCT CCC GTC TGC CCC GGG CGC CTG CCG AGC TAC ATC TAT TTA TAA GCA CGC CAG GGA CTA T	−9.89
	GzmB-17	TGA CAC CGT ACC TGC TCT CGT TGC GTG GGG TTT AGA GTG GGT AGG GAC GCC GGT CAG AAA GCA CGC CAG GGA CTA T	−10.60
	Tr-GzmB	GCT CTC CCG TCT GCC CCG GGC GCC TGC CGA GCT ACA TCT ATT TAT AAG CAC GCC AGG G	−9.89
Perforin	PRF-5	ATA GTC CCT GGC GTG CTT ATA AAT AGA TGT AGC TCG GCA GGC GCC CGG GGC AGA CGG GAG AGC AGG TAC GGT GTC A	−15.21
	PRF-20	TGA CAC CGT ACC TGC TCT GAC ACC GTA CCT GCT CTA TAG TCC CTG GCG TGC TTA AAG AGC AGG TAC GGT GTC A	−23.74
	Tr-PRF	TGC TCT GAC ACC GTA CCT GCT CTA TAG TCC CTG GCG TGC TTA AAG AGC AGG	−8.61

Table S2. Evaluation the docking performance of Tr-GzmB aptamer with target.

Rank	Tr-GzmB aptamer docking model		
	**Docking score**	**Ligand RMSD (Å)**	**Confidence**
1	−301.53	36.02	0.9539
2	−300.35	48.81	0.9529
3	−299.23	49.39	0.9519
4	−287.78	47.94	0.9402
5	−283.37	54.24	0.9351
6	−281.98	41.99	0.9334
7	−280.90	53.21	0.9320
8	−276.19	55.15	0.9258
9	−273.73	55.49	0.9223
10	−273.36	35.43	0.9218

Table S3. Evaluation the docking performance of Tr-PRF aptamer with target.

Rank	Tr-PRF aptamer docking model		
	**Docking score**	**Ligand RMSD (Å)**	**Confidence**
1	−336.92	86.67	0.9768
2	−336.02	91.33	0.9763
3	−330.78	91.50	0.9738
4	−330.00	89.42	0.9734
5	−324.93	85.51	0.9706
6	−315.48	57.91	0.9648
7	−311.96	89.91	0.9623
8	−304.23	89.58	0.9563
9	−304.18	57.99	0.9562
10	−303.51	57.55	0.9556

Table S4. The $\Delta\psi$ values measured by the DP-SIS sensor for each target concentration.

Target	Concentration (fg mL^{-1})	Concentration (fM)	$\Delta\psi$ values (Using original aptamer)	$\Delta\psi$ values (Using Tr-aptamer)
GzmB	1	3.68×10	0.001506	0.001828
	10	3.68×10^2	0.003845	0.004701
	10^2	3.68×10^3	0.006090	0.007127
	10^3	3.68×10^4	0.008412	0.009438
	10^4	3.68×10^5	0.010209	0.011991
	10^5	3.68×10^6	0.012431	0.014556
PRF	1	1.69×10	0.001676	0.002116
	10	1.69×10^2	0.004034	0.005294
	10^2	1.69×10^3	0.006754	0.008395
	10^3	1.69×10^4	0.009308	0.011302
	10^4	1.69×10^5	0.011647	0.014269
	10^5	1.69×10^6	0.014004	0.017334

Table S5. Clinical information of COPD patients. ND means not determined.

Patient number	Type	Gender	Age	Smoking history	FVC (%)	FEV_1 (%)	FEV_1 FVC^{-1} (%)	mMRC	CAT	Complication
1	Plasma	F	92	Never	60	74	90	1	13	ND
2	Plasma	M	85	Never	77	57.8	74	1	14	ND
3	Plasma	M	83	Smoker	64	72	74	3	21	Lung cancer
4	Plasma	F	62	Smoker	71	53	45	1	28	ND
5	Plasma	F	75	Smoker	71	59	56	3	23	Lung cancer
6	Plasma	M	80	Smoker	71	71	68	1	11	Lung cancer
7	Plasma	M	74	Smoker	71	53	45	3	16	Lung cancer
8	Plasma	M	69	Smoker	73	68	66	1	10	ND
9	Plasma	M	75	Smoker	89	74	62	1	11	ND
10	Plasma	M	69	Smoker	53	67	74	0	23	Lung cancer
11	Plasma	M	64	Smoker	61	42.7	72	2	20	Lung cancer
12	Plasma	F	85	Smoker	51	33	49	3	12	ND
13	Plasma	M	70	Smoker	70	40	31	4	25	Lung cancer
14	Plasma	M	79	Ex-smoker	65	45.5	69	2	21	Lung cancer
15	Plasma	M	92	Ex-smoker	85	45	38	2	19	ND
16	Plasma	M	71	Ex-smoker	54	45	48	3	23	ND
17	Plasma	M	68	Ex-smoker	65	37	42	2	19	ND
18	Plasma	F	64	Smoker	67	41	43	2	16	ND
19	Plasma	M	68	Ex-smoker	68	39	38	3	22	ND
20	Plasma	M	77	Never	49	35	36	3	17	ND
21	Plasma	M	63	Ex-smoker	41	41	74	3	24	Lung cancer

Table S6. Inter-chip CV of target detection stability test.

Target sensing condition	Day	Detection signal	Inter-chip CV (%)
4 ℃	0	−0.0888 ± 0.0071	7.9955
	1	−0.0887 ± 0.0046	5.1860
	2	−0.0900 ± 0.0074	8.2222
	3	−0.0862 ± 0.0000	0.0000
	4	−0.0875 ± 0.0071	8.1143
	5	−0.0887 ± 0.0046	5.1860
25 ℃	0	−0.0888 ± 0.0071	7.9955
	1	−0.0888 ± 0.0071	7.9955
	2	−0.0875 ± 0.0064	7.3143
	3	−0.0900 ± 0.0052	5.7778
	4	−0.0887 ± 0.0074	8.3427
	5	−0.0887 ± 0.0071	8.0045
